# Impact of intensive care unit relocation on the transmission dynamics of carbapenem-resistant *Acinetobacter baumannii*: a genetic epidemiology study

**DOI:** 10.3389/fcimb.2026.1729472

**Published:** 2026-03-16

**Authors:** Qiannan E, He Wang, Yan Wang, Keke Li, Qingfeng Shi, Ling Cai, Yinghua Zhang

**Affiliations:** 1 Department of Infection Management, Gansu Provincial Hospital, Lanzhou, China; 2 Department of Anorectal Surgery, Gansu Provincial Hospital, Lanzhou, China; 3 Department of Clinical Laboratory Medicine, Gansu Provincial Hospital, Lanzhou, China; 4 Department of Infection Management, Shanghai, China

**Keywords:** carbapenem-resistant *Acinetobacter baumannii*, healthcare-associated infection, intensive care unit, molecular epidemiology, whole-genome sequencing

## Abstract

**Introduction:**

Intensive care unit (ICU) relocation provides a unique opportunity to assess the impact of environmental renewal on the transmission of multidrug-resistant organisms (MDROs). This study aimed to utilize whole-genome sequencing (WGS) combined with epidemiological data to trace changes in the infection rate and transmission routes of carbapenem-resistant *Acinetobacter baumannii* (CR*Ab*) during ICU relocation, and to evaluate the concurrent implementation of infection control measures.

**Methods:**

Clinical and environmental samples were prospectively collected from a tertiary care hospital in China across three phases: pre-relocation, post-relocation, and post-intervention. Antibiotic susceptibility testing of CR*Ab* isolates was performed using the Kirby-Bauer disk diffusion method and the VITEK-2 system. WGS was performed on all isolates. A phylogenetic tree was constructed based on core-genome single nucleotide polymorphisms (SNPs), and potential transmission chains were inferred. Infection prevention and control indicators for MDROs were also monitored.

**Results:**

A total of 11 CR*Ab* isolates were collected, comprising 10 from patient clinical samples and one from the surface of a disinfected mattress. All isolates demonstrated highly similar antimicrobial resistance profiles and carried a core set of resistance genes, including *bla*OXA-23, *bla*OXA-66, *bla*ADC-73, ant (3'')-IIa, *adeB*, *adeG*, and *adeJ*, with some also harboring *bla*TEM-1. All CR*Ab* isolates as sequence type 2 (ST2). Core-genome SNP phylogenetic analysis clustered the 11 isolates into two clades: Clade 1 contained three isolates, and Clade 2 contained eight isolates. This clustering was consistent with the distribution of resistance genes, and two possible transmission chains were constructed. Over the three-month period surrounding the ICU relocation, the CR*Ab* infection rate exhibited a decreasing trend, hand hygiene compliance improved gradually, and adherence to MDRO isolation protocols increased significantly following interventions by the infection control department (*P* < 0.05).

**Conclusions:**

Although ICU relocation contributed to a reduction in CR*Ab* infection rates through environmental renewal, it did not completely interrupt transmission. WGS analysis integrated with epidemiological data suggested that environmental contamination and patient carriage likely played critical roles in CR*Ab* transmission. Enhanced environmental cleaning and disinfection, improved hand hygiene compliance, and strict isolation of infected patients are crucial for the effective control of MDRO spread.

## 1 Introduction

Healthcare-associated infections (HAIs), particularly those caused by multidrug-resistant organisms (MDROs), pose a major challenge in intensive care units (ICUs) worldwide, significantly increasing patient morbidity and mortality and exacerbating the burden on healthcare (Magiorakos et al., 2012; Serra-Burriel et al., 2020; De Waele et al., 2020; Li et al., 2021). Among these pathogens, carbapenem-resistant *Acinetobacter baumannii* (CR*Ab*) has been listed by the World Health Organization (WHO) as one of the top priority drug-resistant pathogens (Dubey et al., 2025). Its remarkable capacity for environmental persistence, potential for clonal dissemination, and extensive resistance to commonly used antimicrobial agents (including β-lactams, aminoglycosides, and fluoroquinolones), especially the global prevalence of high-risk clones such as sequence type 2 (ST2), make it a major challenge in hospital infection prevention and control (IPC) (Magiorakos et al., 2012; Lurie-Weinberger et al., 2025). With very limited therapeutic options available for CR*Ab* infections, implementing precise and effective IPC strategies is paramount to interrupting its transmission (Kubin et al., 2025). Currently, the control of CR*Ab* and other MDRO transmission in healthcare settings primarily relies on integrated measures such as environmental cleaning, hand hygiene, and patient isolation (Tomczyk et al., 2019). However, conventional epidemiological methods like antibiotic susceptibility testing (AST) profiling and pulsed-field gel electrophoresis (PFGE) typing have limited resolution, which impedes the accurate distinction of highly homologous clones and precise tracing of transmission routes, ultimately hindering the identification of infection sources and transmission chains. The application of whole-genome sequencing (WGS) has brought revolutionary advances to molecular epidemiological studies by utilizing single nucleotide polymorphisms (SNP) analysis to reveal pathogen transmission dynamics and microevolutionary relationships, providing robust evidence for the identification of environmental reservoirs and cross infections events (Purushothaman et al., 2022; Landman et al., 2024; Way et al., 2024).

The complete relocation of an ICU represents a major intervention. By facilitating a thorough environmental renewal and process re-engineering, it provides a unique “natural experiment” scenario for investigating the transmission dynamics of CR*Ab*. Although previous report has indicated that relocation can reduce healthcare-associated infection rates in the short term (Li et al., 2017) and even decrease the detection rate of resistant bacteria (Kim et al., 2018; Tran-Dinh et al., 2018), its impact on the molecular transmission patterns of resistant clones remains poorly defined. It is particularly noteworthy that drug-resistant strains genetically related to those from the original epidemic region may still be identified in the new environment; however, whether their presence results from residual contamination, patient carriage, or human-mediated transmission requires further investigation using high-resolution molecular typing techniques.

Therefore, taking advantage of the unique “natural experiment” scenario presented by the relocation of an ICU to a new building, this study utilized a prospective design that integrated clinical epidemiological data with WGS-based molecular typing. The primary aims were to: (1) precisely trace the clonal transmission dynamics of CR*Ab* across the pre-relocation, post-relocation, and enhanced infection control intervention phases; (2) identify potential environmental reservoirs and assess their transmission risk. Additionally, the implementation and impact of key infection control measures, such as hand hygiene, isolation compliance, and environmental cleaning, were examined within the context of CR*Ab* transmission pathways. The findings of this study are expected to provide a critical evidence base for formulating targeted and effective infection control strategies during future unit renovations or relocations in healthcare institutions.

## 2 Methods

### 2.1 Ethical approval

This study was approved by the Institutional Review Board (IRB) of Gansu Provincial Hospital (Approval No: [2025-628]). Written informed consent according to the Helsinki statement was obtained from patients or families.

### 2.2 Study design and setting

This prospective observational study was conducted in the ICU of a provincial tertiary care hospital in China. The entire ICU was relocated to a newly constructed, previously unoccupied building on July 30, 2024. To systematically evaluate the impact of the relocation event and subsequent infection control interventions on CR*Ab* transmission, the study was divided into three distinct phases: the pre-relocation phase (July 2024), the post-relocation phase (August 2024), and the post-intervention phase (September 2024), following the implementation of sustained infection control measures. The study aimed to track the dynamic changes in the CR*Ab* infection rate, delineate its transmission pathways, and comprehensively evaluate the actual effectiveness of environmental renewal and infection prevention and control measures.

### 2.3 Sample collection and inclusion criteria

#### 2.3.1 Patient data collection

Hospitalization data were prospectively collected for all ICU patients with CR*Ab* infections during the study period. Data included admission time, length of stay, bed utilization, and infection diagnosis date. The inclusion criteria were as follows: all CR*Ab*-infected patients were included in the pre-relocation phase; for the post-relocation and sustained intervention phases, only CR*Ab*-infected patients with an ICU length of stay ≥ 48 hours were included. Patients identified with CR*Ab* within 48 hours of admission were excluded to focus on HAIs.

#### 2.3.2 CR*Ab* isolate storage

All CR*Ab* clinical isolates meeting the inclusion criteria were collected and stored for subsequent WGS analysis.

#### 2.3.3 Environmental surveillance

Environmental sampling was performed monthly using sterile sponge swabs on high-touch surfaces within patient care areas (e.g., healthcare workers’ hands, bedside tables, bed rails, ventilator panels, door handles, and terminally disinfected mattresses). All samples underwent microbial culture and identification. Only samples confirmed positive for CR*Ab* were subjected to WGS.

#### 2.3.4 Evaluation of cleaning efficacy using fluorescent marking

The fluorescent marker method was applied to high-touch surfaces in the bed units of patients infected or colonized with MDROs (e.g., bed rails, bedside tables, and light switches). The qualification rate of cleaning efficacy was assessed on the following day.

#### 2.3.5 Audit of ward isolation practices

Upon receiving a positive MDRO report, the Hospital Infection Control Department immediately conducted unannounced on-site audits in the ward to evaluate the implementation of isolation measures. Following the initial audit, feedback and corrective recommendations were provided to the ward unit. A follow-up on-site audit was performed 24 hours later. Adherence to isolation protocols was calculated monthly for each intervention time point.

#### 2.3.6 Monitoring of hand hygiene compliance

Hand hygiene practices among all ICU healthcare workers (including physicians, nurses, and nursing assistants) were monitored using a covert observation method. The hand hygiene compliance rate was calculated monthly.

### 2.4 Changes in infection control metrics over the surveillance period

The following infection control measures were implemented and monitored as integral components of the prospective surveillance design, allowing their influence on CR*Ab* transmission to be assessed alongside molecular and epidemiological data.

#### 2.4.1 Staff training

Systematic training on the prevention and control of MDRO infections was conducted for all ICU staff (including incumbent and newly hired personnel) during two distinct phases: prior to the ICU relocation and during the sustained intervention phase following relocation.

#### 2.4.2 Patient management

Prior to relocation, efforts were made to discharge or transfer all eligible patients from the unit. For the small number of critically ill patients who could not be transferred before the relocation date, dedicated personnel directly transported them to the new ICU unit on the day of relocation. These patients were uniformly placed in single-occupancy rooms for isolation.

#### 2.4.3 Environmental and equipment management

The relocated ICU unit was newly constructed and occupied for the first time. The majority of medical equipment within the unit was newly procured. Only a limited number of essential non-disposable devices from the old unit were migrated for continued use. All such migrated equipment underwent stringent terminal disinfection, strictly adhering to manufacturer guidelines and hospital infection control protocols, before being introduced into the new unit.

### 2.5 Microbiological analysis for molecular epidemiological investigation

All presumptive Acinetobacter isolates obtained from clinical and environmental samples were identified to the species level using the automated microbial mass spectrometry system (VITEK^®^ MS), confirming *Acinetobacter baumannii*. AST was performed using the Kirby-Bauer disk diffusion method and the VITEK^®^ 2 system to determine the minimum inhibitory concentrations (MICs) for various antimicrobial agents. Results were interpreted according to the guidelines provided by the Clinical and Laboratory Standards Institute (CLSI) document M100-S28 (2018). Multidrug-resistant *Acinetobacter baumannii* (MDRO-*Ab*) was defined as isolates exhibiting non-susceptibility (resistant or intermediate) to at least one agent in three or more of the following antimicrobial categories: penicillin/cephalosporin in combination with β-lactamase inhibitors, carbapenems, aminoglycosides, fluoroquinolones, folate pathway inhibitors, and tetracyclines. CR*Ab* was specifically defined as isolates resistant to imipenem and/or meropenem.

### 2.6 Whole genome sequencing

Genomic DNA was extracted from CR*Ab* isolates using the TIANamp Bacterial DNA Kit (Tiangen, Beijing, China) according to the manufacturer’s instructions. The extracted DNA was fragmented via ultrasonication. Sequencing libraries with an insert size of approximately 500 bp were prepared using the Illumina Nextera DNA Library Preparation Kit, following the manufacturer’s protocol. All libraries were sequenced on an Illumina X-TEN platform (San Diego, CA, USA). Raw sequencing reads were processed by removing adapter sequences and trimming low-quality bases from the read ends. Subsequent quality filtering was performed using a 4-bp sliding window, discarding reads with an average quality score below 15, to obtain high-quality clean data for downstream analysis. MLST was performed on the genomes using the software mlstfinder (v2.23.0). The average sequencing depth was >100× for all isolates. Raw reads were assembled *de novo* using SPAdes (v3.15.5), and assemblies with an N50 > 50 kb and contamination check passed via CheckM (v1.2.2) were retained for downstream analysis.

### 2.7 Single nucleotide polymorphism and phylogenetic analysis

To identify high-confidence SNP sites for phylogenetic reconstruction, reads were mapped to the reference genome using BWA-MEM (v0.7.17). SNP calling was performed using SAMtools/BCFtools (v1.17) with parameters ‘-Q 30 -d 10’. Only core SNPs (present in all isolates) with a minimum mapping quality of 30 and a minimum allele frequency of 90% were retained. Isolates were considered closely related/clonal if separated by ≤10 core SNPs, a threshold commonly used for recent transmission investigations in A. baumannii. The genome of *Acinetobacter baumannii* strain ATCC 19606 (GenBank accession no. CP045110.1) was used as the reference sequence. Sequencing data from each sample were aligned to this reference genome. High-confidence SNP sites were identified with a minimum coverage depth of 10×, and mutation profiles for each isolate were subsequently extracted. Based on these high-quality SNP sites, a phylogenetic tree was constructed using MEGA software (v12.0) under the General Time Reversible model with Gamma distribution (GTR+G). Branch support was assessed with 1000 bootstrap replicates. Furthermore, a SNP heatmap illustrating the distribution of SNPs across genomic regions common to all isolates was generated using TBtools software. The potential transmission pathways of CR*Ab* were inferred by integrating these molecular data with the temporal and spatial information of sample collection.

### 2.8 Statistical analysis

Data analysis was performed using IBM SPSS Statistics version 25.0. Categorical data are presented as frequency (percentage). Group comparisons for categorical variables were conducted using the Chi-square test; if more than 20% of cells had an expected frequency of less than 5 or any expected frequency was less than 1, Fisher’s exact test was employed instead. When the overall test was significant and involved multiple group comparisons, the Bonferroni correction was applied for *post hoc* pairwise comparisons. Continuous data with a non-normal distribution are expressed as median (interquartile range, IQR). Comparisons across multiple groups for such data were performed using the Kruskal-Wallis H test, while the Mann-Whitney U test was used for comparisons between two groups. All hypothesis tests were two-tailed, and a significance level of α = 0.05 was adopted.

## 3 Results

### 3.1 CR*Ab* infection description and spatiotemporal distribution

During the entire study period, encompassing the pre-relocation, post-relocation, and sustained intervention phases, a total of 11 CR*Ab* isolates were collected. The isolates originated from clinical specimens (sputum, bronchoalveolar lavage fluid, and central venous catheter tips) of 10 patients and one environmental surface sample (a terminally disinfected mattress) from the ICU.

In the pre-relocation phase, one CR*Ab* isolate (1-B) was detected from an environmental sample (mattress), and CR*Ab* was identified in clinical specimens from five patients (1-C to 1-G). Key epidemiological links included: (1) Patients 1-D and 1-E sequentially occupied the same hospital bed. Patient 1-E was admitted to this bed on the same day Patient 1-D was discharged and was found to be CR*Ab*-positive seven days later, confirming a healthcare-associated infection. Epidemiological investigation found no evidence of direct contact with other known CR*Ab* carriers or clear evidence of cross-transmission via healthcare workers. (2) Patient 1-F was the only patient transferred directly from the old ICU to the new ICU. At the time of transfer, there were no other patients with CR*Ab* infection or colonization in the new ICU. (3) Patient 1-G was discharged on the day of relocation and did not enter the new ICU.

**Figure 1:**
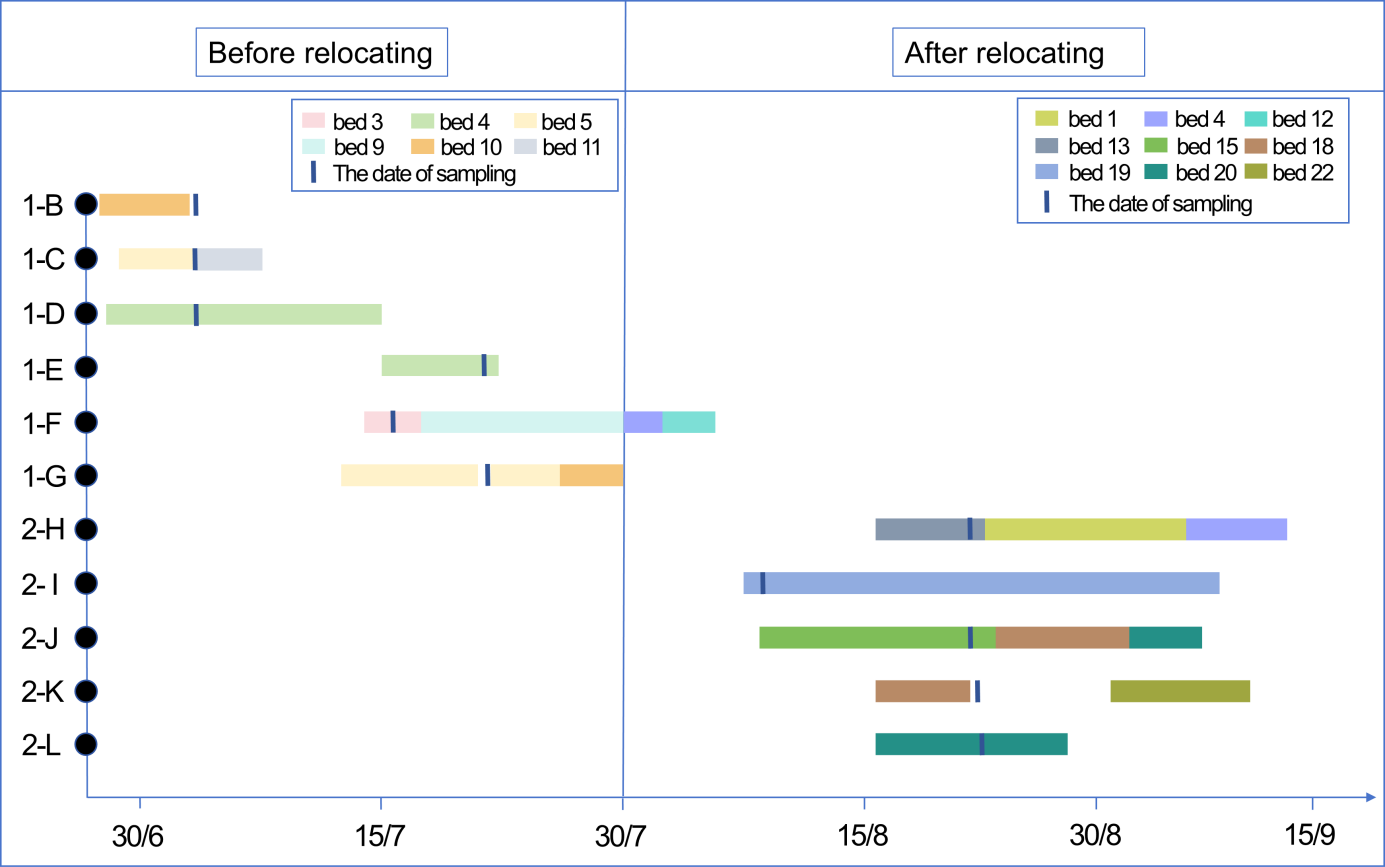
The spatiotemporal distribution map of 11 isolates of CR*Ab*. The rectangular shape indicates the period of hospitalization in ICU, and the same color indicates the same bed. The short vertical line indicates the sampling date. The horizontal axis represents the date, and the vertical axis represents the strain. ICU, intensive care unit; CR*Ab*, carbapenem-resistant *Acinetobacter baumannii*.

In the post-relocation phase, CR*Ab* was detected in clinical specimens from five patients (2-H to 2-L). Among these, Patient 2-H represented the first case of healthcare-associated CR*Ab* infection acquired within the new ICU. No MDROs were detected in any environmental samples during this phase. Following the detection of CR*Ab* in the new ICU, the hospital infection control team immediately initiated and intensified MDRO isolation protocols. Subsequent to these interventions, the rate of CR*Ab* healthcare-associated infections decreased substantially, and no further CR*Ab* was isolated in subsequent environmental sampling. The spatiotemporal distribution, duration of hospitalization, and bed utilization for the patients corresponding to the 11 CR*Ab* isolates are shown in **Figure 1**.

### 3.2 Drug resistance genes and AST

The results of AST are detailed in **Table 1**. The environmental CR*Ab* isolate (1-B) exhibited a highly similar resistance profile to the patient-derived isolates. All 11 isolates demonstrated high-level resistance to carbapenems (meropenem, imipenem). They were universally resistant to β-lactam/β-lactamase inhibitor combinations (including ampicillin/sulbactam, piperacillin/tazobactam, and ticarcillin/clavulanic acid) and exhibited extensive high-level resistance to cephalosporins, aminoglycosides, and fluoroquinolones. All isolates were resistant to doxycycline. However, they remained largely susceptible to trimethoprim-sulfamethoxazole, and with the exception of isolate 2-J, to minocycline and tigecycline. These data indicate that the CR*Ab* strains obtained in this study displayed extensive multidrug resistance, including to cephalosporins, aminoglycosides, and fluoroquinolones. Although minor variations in resistance phenotypes were observed among individual isolates before and after the relocation, no significant changes in the overall resistance rates were noted.

**Table 1:** Drug resistance profiles of carbapenem-resistant *Acinetobacter baumannii* strains.

Strains	MIC																	
	Amikacin	Gentamicin	Tobramycin	Ceftriaxone	Cefotaxime	Cefepime	Ceftazidime	Imipenem	Meropenem	Ampicillin/sulbactam	Ciprofloxacin	Levofloxacin	Minocycline	Doxycycline	Trimethoprim-sulfamethoxazole	Tigecycline	Piperacillin/tazobactam	Ticacillin/clavulanic acid
1-B	64(R)	6(R)	≥16(R)	6(R)	6(R)	≥32(R)	≥64(R)	≥16(R)	≥16(R)	6(R)	≥4(R)	≥8(R)	4(S)	≥16(R)	≤1/19(S)	2(S)	≥128(R)	/
1-C	64(R)	6(R)	≥16(R)	6(R)	6(R)	≥32(R)	≥64(R)	≥16(R)	≥16(R)	6(R)	≥4(R)	≥8(R)	4(S)	≥16(R)	≤1/19(S)	2(S)	≥128(R)	/
1-D	≥64(R)	6(R)	≥16(R)	6(R)	6(R)	16(I)	≥64(R)	≥16(R)	≥16(R)	15(S)	≥4(R)	≥8(R)	2(S)	≥16(R)	≤1/19(S)	2(S)	≥128(R)	≥128(R)
1-E	≥64(R)	6(R)	≥16(R)	6(R)	6(R)	≥32(R)	≥64(R)	≥16(R)	≥16(R)	6(R)	≥4(R)	≥8(R)	4(S)	≥16(R)	≤1/19(S)	2(S)	≥128(R)	≥128(R)
1-F	≥64(R)	6(R)	≥16(R)	6(R)	6(R)	≥32(R)	≥64(R)	≥16(R)	≥16(R)	6(R)	≥4(R)	≥8(R)	4(S)	≥16(R)	≤1/19(S)	2(S)	≥128(R)	/
1-G	≥64(R)	6(R)	≥16(R)	6(R)	6(R)	≥32(R)	≥64(R)	≥16(R)	≥16(R)	6(R)	≥4(R)	≥8(R)	4(S)	≥16(R)	≤1/19(S)	2(S)	≥128(R)	≥128(R)
2-H	≥64(R)	6(R)	≥16(R)	6(R)	6(R)	≥32(R)	≥64(R)	≥16(R)	≥16(R)	11(R)	≥4(R)	≥8(R)	4(S)	≥16(R)	≤1/19(S)	2(S)	≥128(R)	≥128(R)
2-I	≥64(R)	6(R)	≥16(R)	6(R)	6(R)	≥32(R)	≥64(R)	≥16(R)	≥16(R)	6(R)	≥4(R)	≥8(R)	4(S)	≥16(R)	≤1/19(S)	2(S)	≥128(R)	≥128(R)
2-J	≥64(R)	6(R)	≥16(R)	6(R)	6(R)	32(R)	≥64(R)	≥16(R)	≥16(R)	11(R)	≥4(R)	≥8(R)	≥16(R)	≥16(R)	≤1/19(S)	4(I)	≥128(R)	≥128(R)
2-K	≥64(R)	/	≥16(R)	6(R)	6(R)	≥32(R)	≥64(R)	≥16(R)	≥16(R)	6(R)	≥4(R)	≥8(R)	4(S)	≥16(R)	≤1/19(S)	2(S)	≥128(R)	≥128(R)
2-L	≥64(R)	6(R)	≥16(R)	6(R)	6(R)	≥32(R)	≥64(R)	≥16(R)	≥16(R)	6(R)	≥4(R)	≥8(R)	4(S)	≥16(R)	≤1/19(S)	2(S)	≥128(R)	≥128(R)

MIC, Minimum inhibitory concentration; S indicates susceptible, I intermediate, and R resista.

### 3.3 WGS and SNP analysis

**Figure 2:**
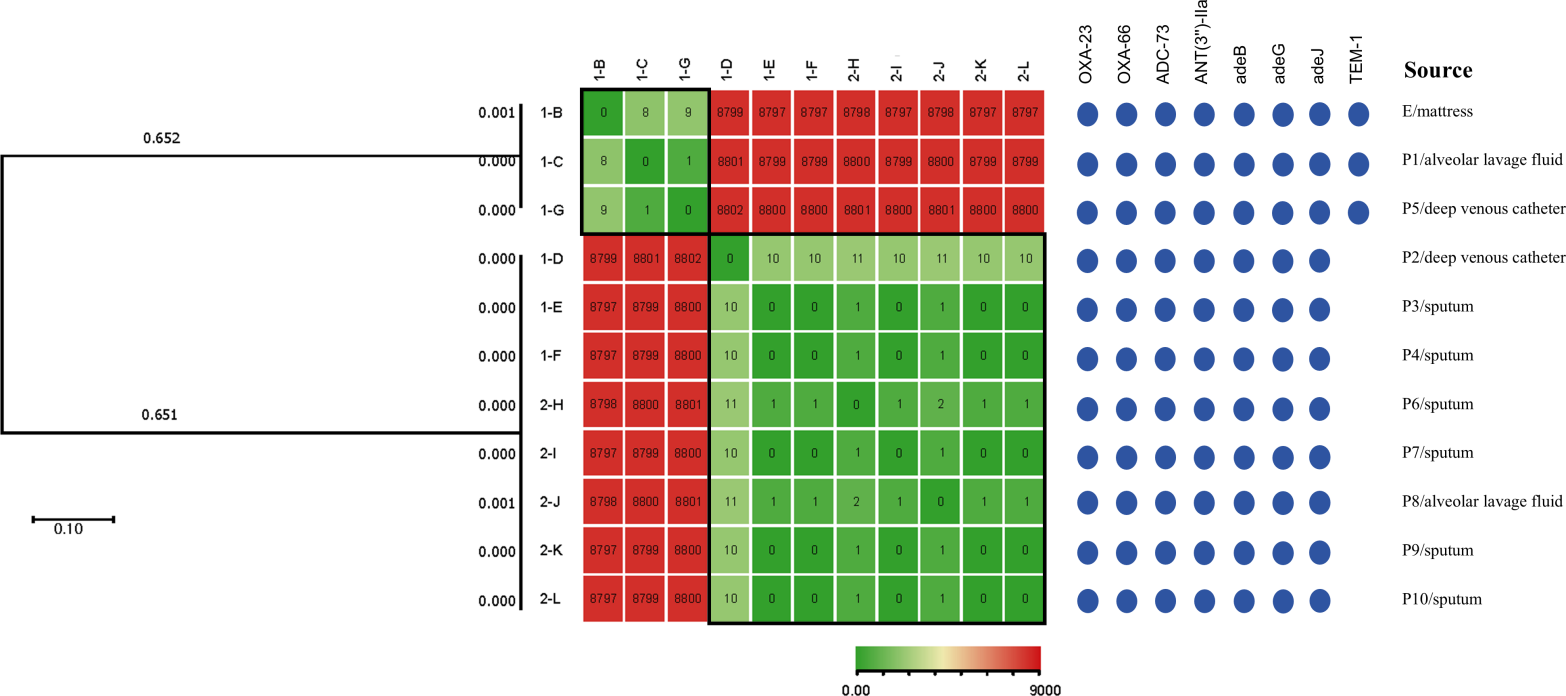
Phylogenetic tree and SNP difference heatmap of the 11 CR*Ab* isolates. The branch length for each isolate represents the degree of evolutionary variation. A shorter cumulative branch length between any two isolates indicates fewer genetic differences and a closer evolutionary relationship. The scale bar for genetic variation is 0.10. The heatmap represents the number of SNP differences, with red indicating a higher number and green a lower number. Black squares highlight homologous sections. Blue circles denote detected characteristic CR*Ab* resistance genes. P, patient; E, environment.

WGS was performed on all 11 CR*Ab* isolates, with confirmatory identification as carbapenem-resistant *Acinetobacter baumannii*. MLST identified all isolates as sequence type 2 (ST2), a globally prevalent high-risk multidrug-resistant clone. Further analysis of resistance genes revealed that all isolates carried the *bla*OXA-23 carbapenemase gene, representing the primary mechanism for their carbapenem resistance. Core-genome SNP phylogenetic analysis, using *Acinetobacter baumannii* ATCC 19606 as the reference, clustered the 11 CR*Ab* isolates into two distinct phylogenetic clades (with pairwise SNP differences of less than 10). Clade 1 comprised three isolates: the environmentally derived mattress isolate (1-B) and patient isolates (1-C and 1-G). Clade 2 contained the remaining eight patient-derived isolates. This phylogenetic clustering was consistent with the distribution pattern of the *bla*TEM-1 resistance gene (**Figure 2**).

**Figure 3:**
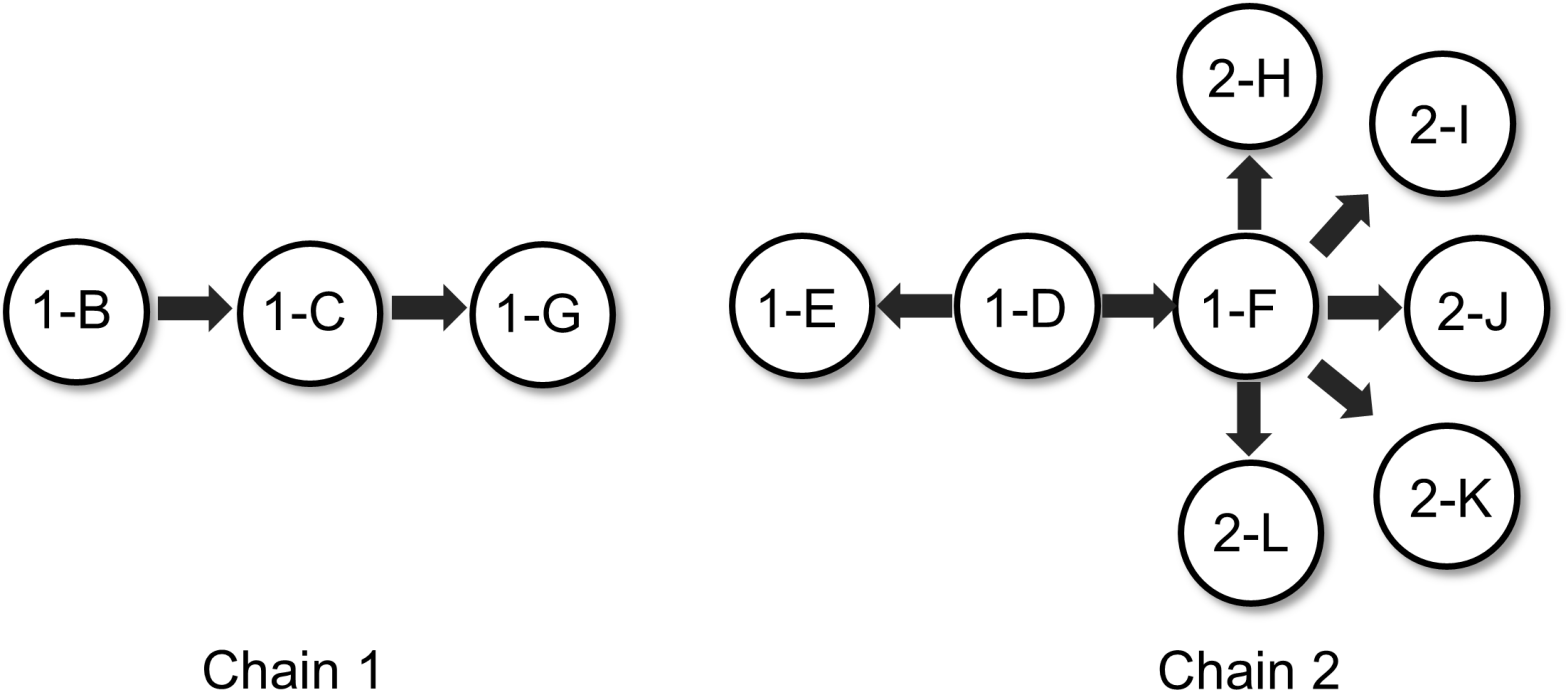
Transmission chain diagram.

By integrating phylogenetic relationships with spatiotemporal epidemiological data, we proposed two plausible transmission chains (**Figure 3**). The clustering in Chain 1 is consistent with environmental vehicle transmission, where the inadequately terminally disinfected mattress (1-B) may have acted as a reservoir linked to patients 1-C and 1-G. The genetic homogeneity within Chain 2, combined with bed turnover and transfer events, supports the possibility of patient-to-patient transmission, likely facilitated by the sequential occupancy of the same bed (between patients 1-D and 1-E) and cross-infection during concurrent hospitalization (between patients 1-D and 1-F). This was followed by the subsequent introduction of CR*Ab* into the new ICU unit by patient 1-F, which appears to have led to secondary infections in patients 2-H through 2-L.

### 3.4 Evaluation of infection control efficacy before and after relocation

As shown in **Table 2**, this study demonstrated a significant downward trend in the overall MDRO infection rate during the three months encompassing the ICU relocation and subsequent sustained intervention period (*P* < 0.05). Correspondingly, the CR*Ab* infection rate also decreased markedly. Inter-group comparisons revealed a statistically significant difference in the infection rate between September and July (*P* < 0.05).

**Table 2:** Analysis of infection control indicators from July to September.

Variable	July	August		September	*χ²/H*	*P-value*
MDRO infection rate, n/N (%)	10/76 (13.2)^a^	8/124 (6.5)^a^		4/106 (3.8)^a^	6.012^*^	0.049
CR*Ab* infection rate, n/N (%)	7/76 (9.2)^a^	6/124 (4.8)^a,b^		0/106 (0)^b^	^*^	0.003
Hand hygiene compliance rate, n/N (%)	33/46 (71.7)^a^	54/67 (80.6)^a^		138/140 (98.6)^b^	31.761^*^	0.000
Fluorescence labeling clearance rate, n/N (%)	50/55 (90.9)	40/48 (83.3)		60/78(76.9)	4.455^*^	0.108
Environmental microbial qualification rate, n/N (%)	45/55 (81.8)	24/28 (85.7)		14/22(63.6)	4.161^*^	0.125
MDRO isolation measures rate, Median (IQR) [%]	91.18 (10.3)	94.12(29.41)		94.12(23.53)	1.778^#^	0.411
Paired Comparison						
Variable	First observation		Second observation		*Z*	*p-value*
MDRO isolation measures rate, Median [IQR] [%]	82.35 (23.53)		100 (5.88)		-4.399^Δ^	0.000

*Chi-square test; ^#^Kruskal-Wallis H test; ^Δ^Mann, Whitney U test. Values with superscript letters a, b are significantly different across columns (P<0.05).

Regarding infection control metrics, hand hygiene compliance rate increased progressively each month (*P* < 0.05), with the rate in September being significantly higher than those in both July and August (*P* < 0.05). Although the fluorescent marker clearance rate showed minor monthly fluctuations and the environmental microbiological qualification rate exhibited an initial increase followed by a decrease, these changes were not statistically significant (**Table 2**).

When assessed on a monthly basis, the compliance rate with MDRO isolation protocols showed an increase in August and September compared to July; however, this difference did not reach statistical significance (*P* > 0.05). In contrast, compliance rates measured during unannounced audits conducted 24 hours apart demonstrated a significant improvement in adherence following the intervention and feedback session compared to the pre-intervention assessment (*P* < 0.05). This indicates that immediate feedback and corrective measures can effectively enhance the quality of isolation practice implementation (**Table 2**).

## 4 Discussion

The complete relocation of an ICU represents a potential intervention capable of altering the transmission dynamics of MDROs. This study, by integrating WGS with prospective epidemiological surveillance, elucidated the persistent transmission of a high-risk ST2 CR*Ab* clone within the unique context of a purported “environmental reset”. Our results demonstrate that even under conditions of complete infrastructural renewal, CR*Ab* transmission can persist and disseminate through cryptic environmental reservoirs and patient-to-patient cross-transmission. WGS analysis successfully reconstructed two distinct transmission chains and confirmed that reinforcing infection control measures, particularly improving adherence to isolation protocols, was pivotal in ultimately containing the outbreak.

A total of 11 CR*Ab* isolates were obtained from patient clinical specimens and ICU environmental samples in this study. MLST identified all isolates as ST2, consistent with the distribution of the predominant CR*Ab* clone observed globally (Liu et al., 2022; Baleivanualala et al., 2023), further confirming the significant adaptability and transmission advantage of this clonal lineage in healthcare settings. All isolates harbored the *bla*OXA-23 carbapenemase gene, representing the primary molecular basis for their carbapenem resistance (Wu et al., 2016). Most importantly, phylogenetic analysis based on core-genome SNPs revealed that all isolates could be segregated into two distinct clades, with pairwise SNP differences of less than 10. This strongly suggests that all isolates originated from a recent common ancestor, indicating a single clonal complex responsible for localized transmission within the hospital, rather than multiple independent introduction events. This finding highlights a potential advantage over traditional typing methods, such as pulsed-field gel electrophoresis (PFGE), which might fail to differentiate these closely related strains. This highlights the critical evidence provided by WGS-based molecular tracing for identifying key transmission links and evaluating the effectiveness of infection control measures (Leopold et al., 2014; Sherry et al., 2022).

By integrating spatiotemporal epidemiological data, this study successfully inferred two distinct transmission chains. The clustering of the environmentally derived strain (mattress, 1-B) with patient isolates (1-C, 1-G) in Clade 1 strongly suggests that inadequately terminally disinfected environmental surfaces can act as a transmission reservoir. Furthermore, the high degree of homology among all isolates within Clade 2, which included a key patient (1-F) transferred directly from the old ICU to the new unit, indicates that this individual likely served as a “bridge case” introducing CR*Ab* into the new ward, subsequently seeding further transmission. Although the overall infection rate decreased significantly post-relocation, confirming the short-term beneficial effect of environmental renewal, the intervention failed to completely eliminate all reservoirs of transmission, particularly certain residual non-disposable equipment and infected or colonized patients. This finding provides a plausible explanation for the persistence of transmission events within the new unit.

This study found that although the monthly aggregate compliance rate with isolation protocols showed no significant difference, unannounced audits conducted 24 hours apart revealed a significantly higher adherence rate post-intervention compared to pre-intervention. These findings underscore that real-time monitoring and feedback, conducted as part of the transmission surveillance framework, may enhance the implementation of control measures, thereby supporting the interruption of CR*Ab* dissemination. Furthermore, the monthly increase in hand hygiene compliance correlated with the declining trend in CR*Ab* infection rates, underscoring its role in interrupting transmission. Although the qualification rate of fluorescent marker cleaning did not show a significant change, reflecting instances of untimely and incomplete cleaning and disinfection, the subsequent immediate feedback and corrective actions mitigated the associated risk of transmission. This highlights the critical importance of the timely identification and management of risk points for effective prevention and control. The absence of a significant difference in environmental microbiological qualification rates may be related to variations in sampling timing, which was not consistently performed immediately post-cleaning and disinfection. This suggests the future implementation of more standardized monitoring protocols.

This study applied WGS technology to the unique “natural experiment” scenario of an ICU relocation. It molecularly confirmed that even implementing an extreme environmental intervention alone is insufficient to eradicate the transmission of CR*Ab*, thereby deepening the understanding of the complex ecology of MDROs within the hospital setting. Furthermore, the research demonstrates the potential value of WGS in real-time hospital infection control. It not only enables the precise identification of critical links in transmission chains, such as specific environmental reservoirs and human transmission nodes, but also provides a technological pathway towards achieving “precision infection control.” Based on these findings, we recommend that future unit relocation or renovation plans incorporate, in addition to environmental upgrades, a heightened focus on active MDRO screening for transferred patients—particularly long-term occupants—and ensure the thorough terminal disinfection of all migrated equipment. The evaluation of infection control efficacy should rely on high-frequency, unannounced audit mechanisms. Concurrently, we suggest that well-resourced hospitals progressively establish WGS technology platforms, integrating them as routine tools for investigating complex outbreaks.

This study has several limitations. First, the observational design at a single center, coupled with the limited number of CR*Ab* isolates (n=11) recovered despite prospective screening, constrains the statistical power and generalizability of our findings. While WGS provides high resolution, the precise directionality of transmission cannot be definitively established without more frequent longitudinal sampling from patients and the environment. Second, although environmental sampling targeted high-touch surfaces, other reservoirs (e.g., air, water, less accessible equipment) were not sampled, potentially missing contributing sources. Third, the infection control interventions were implemented as a bundle; therefore, the individual contribution of each measure (e.g., hand hygiene vs. enhanced cleaning) to the observed decline in CR*Ab* rates cannot be disentangled. Future multi-center studies with larger cohorts and more comprehensive environmental sampling are needed to validate these observations and further elucidate the complex interplay between environmental reset, patient carriage, and infection control measures in shaping CR*Ab* epidemiology.

In conclusion, while upgrading the physical environment can reduce the risk of CR*Ab* transmission to some extent, it is insufficient alone to achieve complete interruption of transmission. Molecular epidemiological investigation supported by WGS has provided insights into the complex cross-transmission network between the environment and patients, underscoring the critical importance of continuously strengthening the monitoring of infection control measures. Future research could be directed towards developing real-time outbreak early warning systems supported by WGS, evaluating the cost-effectiveness of WGS implementation, and exploring more efficient infection prevention and control strategies.

## Data Availability

The raw sequence data presented in this study have been deposited in the Genome Sequence Archive (GSA) of the China National Center for Bioinformation (GSA: CRA039653).
